# CYR61 triggers osteosarcoma metastatic spreading via an IGF1Rβ-dependent EMT-like process

**DOI:** 10.1186/s12885-019-5282-4

**Published:** 2019-01-14

**Authors:** Nadia Habel, Bojana Stefanovska, Dimitri Carène, Ana Patiño-Garcia, Fernando Lecanda, Olivia Fromigué

**Affiliations:** 1 Inserm, UMR981, Gustave Roussy, 39 Rue Camille Desmoulins, F-94805 Villejuif, France; 2 Gustave Roussy, F-94805 Villejuif, France; 3 Université Paris Diderot, F-75013 Paris, France; 4 Université Paris Sud, F-91400 Orsay, France; 5 University of Navarra, Center for Applied Medical Research, E-31008 Pamplona, Spain; 6 Present address: Inserm U1065, Mediterranean Centre for Molecular Medicine, F-06204 Nice, France

**Keywords:** Bone tumor, CCN1, IGF, EMT, MET, Metastasis

## Abstract

**Background:**

Osteosarcoma is the most prevalent primary bone malignancy in children and young adults. These tumors are highly metastatic, leading to poor outcome. We previously demonstrated that Cysteine-rich protein 61 (CYR61/CCN1) expression level is correlated to osteosarcoma aggressiveness in preclinical model and in patient tumor samples. The aim of the present study was to investigate the CYR61-induced intracellular mechanisms leading to the acquisition of an invasive phenotype by osteosarcoma cells.

**Methods:**

Modified murine and human osteosarcoma cell lines were evaluated for cell adhesion, aggregation (spheroid), motility (wound healing assay), phenotypic markers expression (RT-qPCR, western blot). Cell-derived xenograft FFPE samples and patients samples (TMA) were assessed by IHC.

**Results:**

CYR61 levels controlled the expression of markers related to an Epithelial-mesenchymal transition (EMT)-like process, allowing tumor cells to migrate acquiring a competent morphology, and to be able to invade the surrounding stroma. This phenotypic shift indeed correlated with tumor grade and aggressiveness in patient samples and with the metastatic dissemination potential in cell-derived xenograft models. Unlike EGFR or PDGFR, IGF1Rβ levels correlated with CYR61 and N-cadherin levels, and with the aggressiveness of osteosarcoma and overall survival. The expression levels of IGF1Rβ/IGF1 axis were controlled by CYR61, and anti-IGF1 neutralizing antibody prevented the CYR61-induced phenotypic shift, aggregation, and motility abilities.

**Conclusions:**

Taken together, our study provides new evidence that CYR61 acts as a key inducing factor in the metastatic progression of osteosarcoma by playing a critical role in primary tumor dissemination, with a process associated with IGF1/IGFR stimulation. This suggests that CYR61 may represent a potential pivotal target for therapeutic management of metastases spreading in osteosarcoma, in correlation with IGF1/IGFR pathway.

**Electronic supplementary material:**

The online version of this article (10.1186/s12885-019-5282-4) contains supplementary material, which is available to authorized users.

## Background

Osteosarcoma is the most common primary malignant non-hematopoietic bone tumor, particularly affecting adolescents and young adults. These tumors frequently spread locally or to distant organs (predominately to the lungs, brain or other skeletal sites). Patients with localized osteosarcoma are treated by surgery, with neo-adjuvant and adjuvant chemotherapy, and have an event-free-survival (EFS) of 60–70% at 5 years. In contrast, patients with detectable metastases at diagnosis or with recurrent disease show a dismal overall prognosis with 5-year EFS of less than 30% [1]. This major clinical problem precludes the development of new therapeutic strategies able to improve these poor current clinical outcomes.

The immediate-early gene *CYR61* encodes a member of the extracellular matrix-associated CCN family of six homologous cysteine-rich proteins comprising connective tissue growth factor (CTGF), nephroblastoma overexpressed (NOV), and Wnt-induced secreted proteins (WISPs). CYR61 is involved in multiple physiological functions among which skeletal and cardiovascular development and injury repair [2–5]. In different solid tumors, CYR61 was shown to promote tumor growth and vascularization as well as cell invasiveness and metastasis [6–10]. We previously highlighted a positive correlation between CYR61 protein level and osteosarcoma cell dissemination both in vitro and in vivo [11, 12]. CYR61 was able to promote tumor neo-angiogenesis and extracellular matrix remodeling suggesting a potential role in tumor cells dissemination [11, 12]. These in vitro and preclinical observations have been strengthened at a clinical level since CYR61 protein levels were associated with tumor grade in osteosarcoma patients [11, 12]. Thus, metastatic tumor samples express higher levels of CYR61 than localized tumors, and that recurrent tumor tissues exhibit the highest levels of CYR61. Moreover, CYR61 protein levels in osteosarcoma biopsies correlate significantly with poor overall survival of the patients [13]. As a consequence CYR61 may be associated with a metastatic-promoting activity in osteosarcoma. Yet the precise mechanism of action of CYR61 on osteosarcoma cell dissemination ability remains unclear.

A developmental cellular program called Epithelial-to-Mesenchymal Transition (EMT) confers epithelial cancer cells with novel functions including migration, invasion to the surrounding stroma and dissemination to secondary sites, substantiating the progression of early-stage tumor towards a high-grade malignancy [14, 15]. This EMT program comprises the activation of transcription factors (Slug, Snail, Twist, ZEB1…) driving the downregulation or loss of epithelial cell junction markers (E-cadherin…) and the upregulation or gain of mesenchymal markers (N-cadherin, Vimentin…). Many extracellular signals can activate a trans-differentiation program in epithelial cells that leads to EMT [16]. In this context, growth factors such as Hepatocyte Growth Factor (HGF), Fibroblast Growth Factor (FGF), Epidermal Growth Factor (EGF), Platelet-Derived Growth Factor (PDGF), Insulin-like Growth Factor 1 (IGF1) Transforming Growth Factor-β (TGFβ) or Bone Morphogenetic Proteins (BMPs), often induce EMT in epithelial cells through the activation of transmembrane tyrosine kinase receptors (RTKs) [14].

In the resting phase a single layer of osteoblasts cover all bone surfaces creating a histological structure reminiscent of an epithelial-like monolayer. In contrast, transformed cells of osteosarcoma, despite their mesenchymal origin, have recently been reported to undergo a phenotypic switch evocative of an EMT-like process, with the acquisition of an increase invasiveness and motility leading to increased pro-metastatic activity. This event shares several features of the classical EMT observed in solid tumors of an epithelial origin [17–20]. The tumor microenvironment consisting in surrounding stroma plays a key role in osteosarcoma tumorigenesis. Tumor cells are embedded in an intricated network of fibrillar extracellular matrix with contain a rich mixture of growth factors within the bone marrow stroma. TGFβ is the only one reported up to now to promote osteosarcoma invasion and metastasis through the induction of an EMT-like process [21].

The present study reports that CYR61 triggers specific and characteristic features relative to EMT in vitro, in a murine preclinical model and in patient tumor samples. We also report a positive correlation between CYR61 and IGF1Rβ levels and show that CYR61 controls IGF1 and IGF1Rβ expression levels, modulating the related intracellular signaling. Taken together, our data demonstrate the involvement of CYR61 in the early metastatic cascade such as the acquisition of invasive properties by osteosarcoma cells. This reinforces CYR61 as a pivotal factor for the therapeutic management of metastasis in osteosarcoma.

## Results

### CYR61 and N-cadherin expression levels are correlated in osteosarcoma

**Fig. 1:**
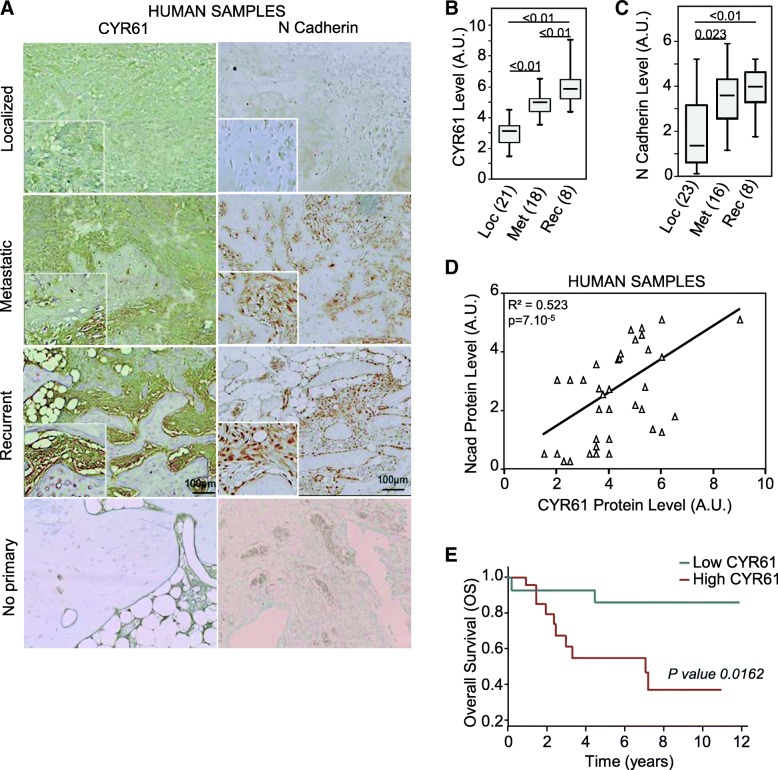
CYR61 controls the levels of N-cadherin in tumor tissues. (**a**) Immunohistochemical staining for N-cadherin and CYR61 of tissue microarray (TMA) containing human samples derived from localized, metastatic or recurrent osteosarcoma. No primary antibody verifies the specificity of the staining and lacked background noise. **b**, **c** Box plot of IHC staining score for CYR61 (**b**) and N cadherin (**c**). Results are expressed as arbitrary units (A.U.) (**d**) Spearman correlation between N-cadherin and CYR61 expression levels in human samples (*n* = 47). The black line shows the regression line. (**e**) Kaplan-Meier survival curves from patients with low CYR61 level or patients with highest CYR61 level.

Tissue microarray (TMA) comprised of 233 osteosarcoma and 28 normal bone core samples (Additional file 1: Figure S1) was used to assess the expression level of CYR61 and N-cadherin (Fig. 1a). The average IHC staining score for N-cadherin and CYR61 increased with tumor aggressiveness: metastatic and recurrent tumor tissues expressed respectively 1.6 and 2 times more N-cadherin or CYR61 than localized primary tumor tissues (Fig. 1b-c). Furthermore, the expression levels of these two markers were positively correlated (Pearson correlation coefficient = 0.523; *p* = 7 × 10^− 4^; Fig. 1d). CYR61 expression was associated with significant decrease in overall survival compared to low expression (*p* = 0.0162; Fig. 1e). A Cox proportional-hazards regression model confirmed that CYR61 up-regulation predicted higher risk of death (*p* = 0.041).

Altogether, these data indicate close correlation between CYR61 and N-cadherin expression level and tumor aggressiveness in osteosarcoma samples.

### CYR61 controls the relative level of N- and E-cadherin in osteosarcoma cells

**Fig. 2:**
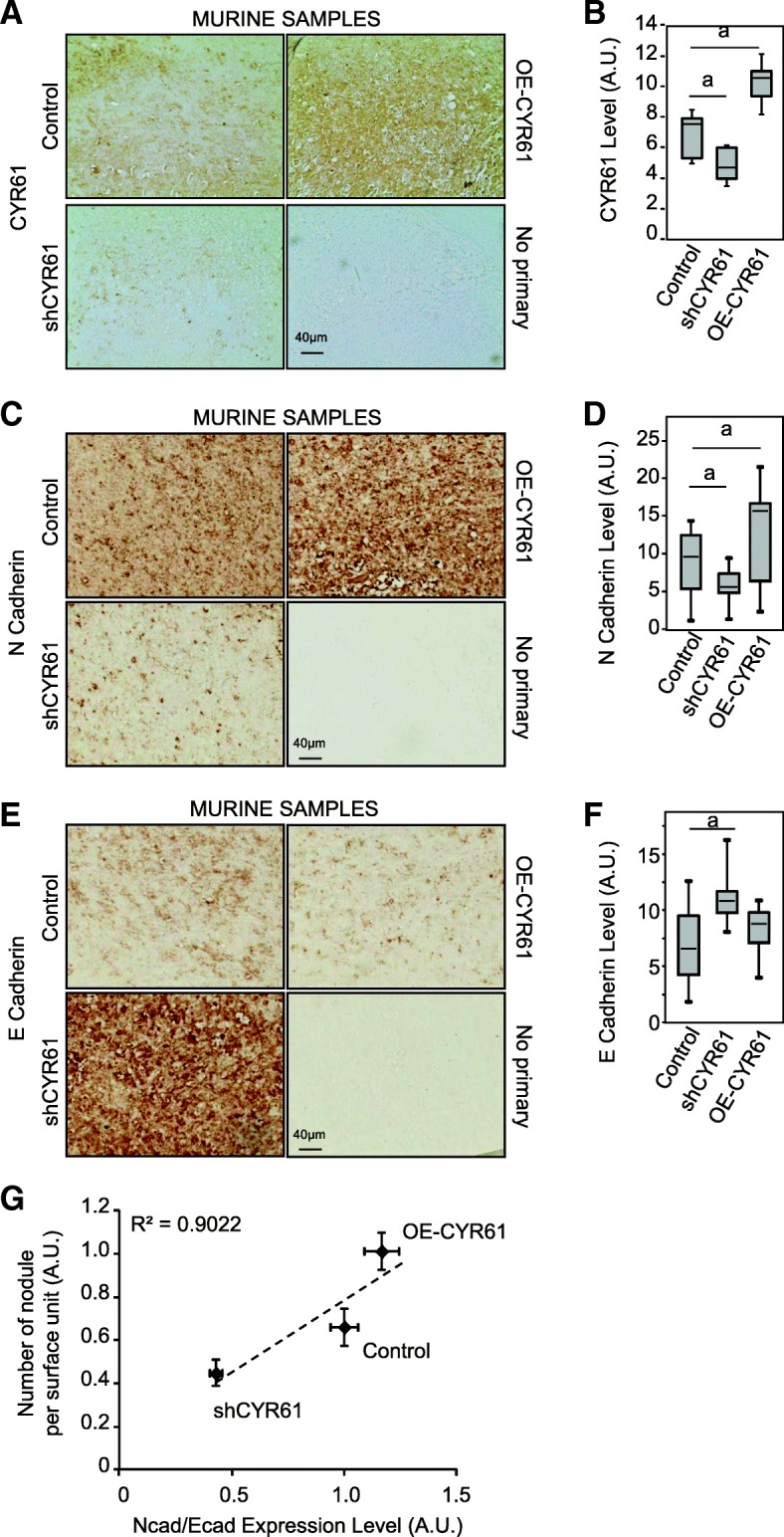
CYR61 controls the relative levels of N-and E-cadherin in tumor tissues. Immunohistochemical staining for CYR61 (**a**), N-cadherin (**c**), and E-cadherin (**e**) of control, CYR61-silenced or CYR61-overexpressing cell-line derived xenografts. Box plot of IHC staining scores for CYR61 (**b**), N-cadherin (**d**), and E-cadherin (**f**). a: *p* < 0.05 vs. control cells. **g** Spearman correlation between the number of metastases, and N−/E-cadherin ratio in murine samples

In order to determine the influence of CYR61 on osteosarcoma cell behavior, new osteosarcoma stable cell lines were generated by lentiviral transduction to silence or overexpress CYR61 (Fig. 4b). Modified and parental cells injected to mice generated primary tumors that expressed various CYR61 levels, as assessed by IHC (Fig. 2a-b). CYR61 silencing led to a reduced expression of N-cadherin (− 34%, *p* = 3 × 10^− 6^, Fig. 2c-d), and an increased expression of E-cadherin compared to control tumor cells (+ 58%, *p* = 2 × 10^− 13^; Fig. 2e-f). Conversely, CYR61 overexpression led to an increased expression of N-cadherin (+ 41%, *p* = 1.3 × 10^− 4^), and a slightly reduced expression of E-cadherin (− 9%, *p* = 0.057). Globally, the N/E-cadherin ratio fully correlated to CYR61 expression levels (Fig. 2g).

**Fig. 3:**
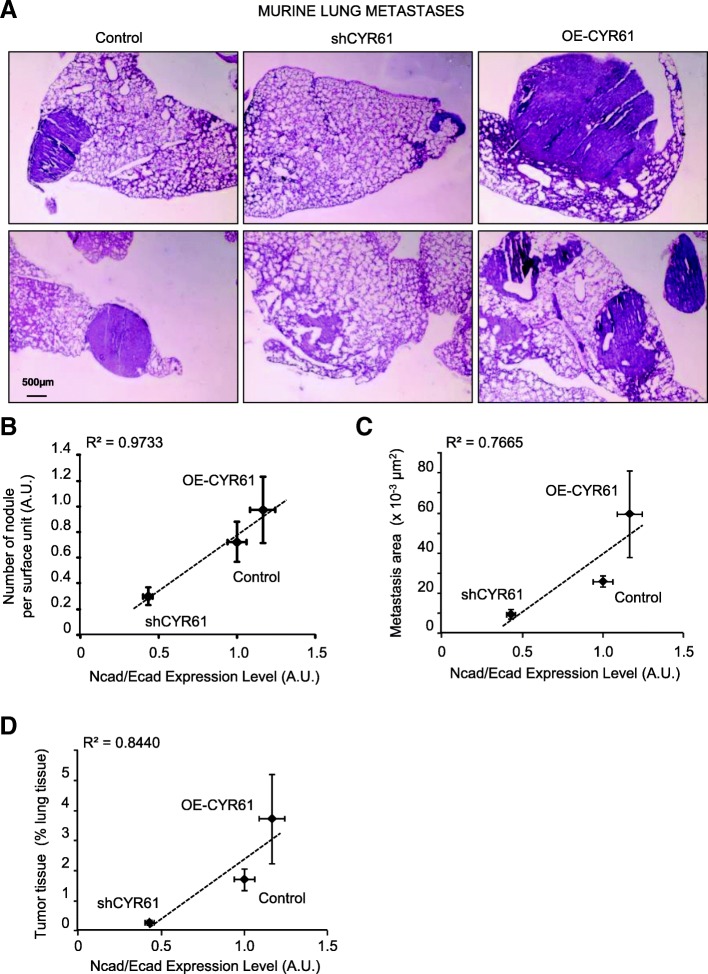
CYR61 controls the osteosarcoma metastatic potential in correlation with the relative levels of N- and E-cadherin. **a** H&E staining of lung tissue sections isolated from mice injected with control, shCYR61, or OE-CYR61 cells. Spearman correlations between the metastatic nodules number (**b**), area of metastases (**c**) or the relative metastatic pulmonary tissue volume (**d**) and N−/E-cadherin ratio

The lung metastatic burden was assessed by HES staining of paraffin-embedded sections (Fig. 3a). The number and size of metastatic nodules positively correlated with CYR61 and with N-cadherin/E-cadherin expression level ratio (Fig. 3b-d).

Altogether, these data indicate that CYR61 controls the relative levels of N- and E-cadherin in osteosarcoma cells. Furthermore, the relative levels of N-, E-cadherin and CYR61 correlate with tumor spread, suggesting the intervention of a process related to the epithelial-to-mesenchymal transition (EMT).

### CYR61 controls the relative expression level of epithelial and mesenchymal markers

**Fig. 4:**
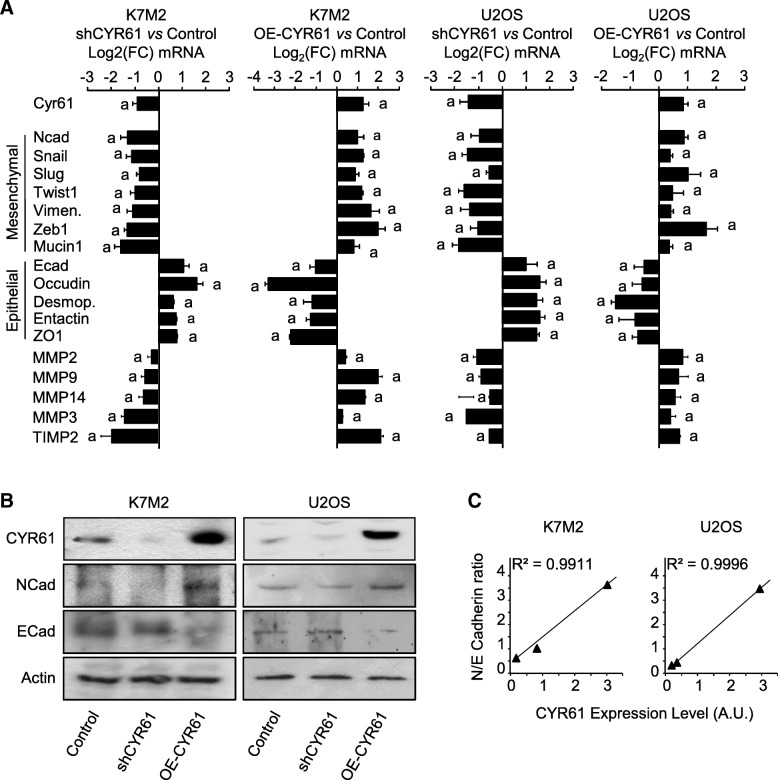
CYR61 controls osteosarcoma cell phenotype in vitro. **a** Expression pattern of a panel of mesenchymal and epithelial markers in K7 M2 and U2OS cell lines, as assessed by RT-qPCR. The relative mRNA levels were calculated using the 2^–ΔΔCT^ method and expressed as Log2 of fold change (mean ± standard deviation). a: *p* < 0.05 vs. control cells. **b** Expression pattern of CYR61, N- and E-cadherin proteins in K7 M2 and U2OS cell lines, as assessed by Western blot. Actin was used as loading control. **c** Spearman correlation between N−/E-cadherin ratio and CYR61 expression level in K7 M2 and U2OS cell lines. The black line shows the regression line

In order to better characterize the molecular mechanisms involved in the pro-metastatic effect of CYR61, gene expression levels of a panel of epithelial and mesenchymal markers were assessed by real time RT-qPCR in both murine K7 M2 and human U2OS modified cell lines (Fig. 4a). As expected, CYR61 silencing led to a reduced expression of N-cadherin and an increased expression of E-cadherin, and vice versa CYR61-overexpression led to an increased expression of N-cadherin and a reduced expression of E-cadherin in both murine K7 M2 and human U2OS cell lines. CYR61 silencing was also associated with a reduced expression of mesenchymal-related markers (Snail-1, Snail-2/Slug, Twist1, Vimentin, ZEB1, Mucin-1) and an increased expression of epithelial-related markers (Occludin, Desmoplakin, Entactin, ZO-1) in murine K7 M2 and human U2OS cell lines. Conversely, CYR61 overexpression led to an increase in gene expression levels of mesenchymal related markers and a decrease in epithelial-related markers. Likewise, modulation of CYR61 expression levels was associated with matrix metalloproteases-2, − 3, − 9, and − 14 and TIMP2 in both K7 M2 and U2OS cell lines.

The protein expression level of N- and E-cadherin was assessed by western blot (Fig. 4b). As expected, CYR61 silencing led to a reduced expression of N-cadherin and an increased expression of E-cadherin. Conversely, CYR61 overexpression led to an increased expression of N-cadherin and a reduced expression of E-cadherin. Globally, the N/E-cadherin ratio fully correlated to CYR61 expression level in murine K7 M2 and human U2OS cell lines (Fig. 4c).

Altogether, these data indicate that CYR61 modulates the expression level of a variety of markers related to epithelial-to-mesenchymal transition (EMT) in osteosarcoma cells.

### CYR61 controls the cell morphology and relative adhesion performance

Control and CYR61-modified osteosarcoma cells, routinely cultured on plastic in serum-supplemented medium, exhibited different morphological characteristics (Additional file 2: Figure S2A). CYR61-silenced cells exhibited a more cuboidal shape than control cells whereas CYR61 over-expressing cells exhibited an elongated and more or less branched shape. As a result, the average maximum cell length was related to CYR61 expression level (Pearson correlation coefficient > 0.99, *p* < 0.05; Additional file 2: Figure S2B).

Furthermore, cells were tested in 3-D conditions for spheroid formation. K7 M2 cells, seeded in poly-Hema coated 96-well round-bottomed plates, reproducibly formed condensed and organized 3D structures in less than 24 h (Additional file 2: Figure S2C-D). CYR61 silencing lowered osteo-spheroid growth whereas CYR61 overexpression markedly increased osteo-spheroid growth and cell invasion. The migration of cells out of the spheroid into the close Matrigel was also clearly favored for cells overexpressing CYR61, and markedly reduced for CYR61-silenced cells. Using this 3D assay as a more physiologically predictive cancer invasion model, our results confirmed that CYR61 play a key role in osteosarcoma cell invasiveness.

The osteosarcoma cells ability to interact with the extracellular matrix was evaluated using plastic surface coated or not with some key bone matrix components (Additional file 2: Figure S2E). The cell adhesion rate onto uncoated plastic or laminin- or fibronectin-coated surfaces was not dependent on CYR61 level. In contrast, cell adhesion onto type I collagen was accelerated in silenced cells and slowed in CYR61-overexpressing cells.

Altogether, these modulations of cell fate also suggest a CYR61-dependent balance between mesenchymal and epithelial-like phenotypes. Subsequent adhesive properties, both at cell-cell and cell-matrix levels, are dependent on CYR61 levels.

### CYR61 expression level correlates with that of IGF1Rβ, not EGFR or PDGFR

**Fig. 5:**
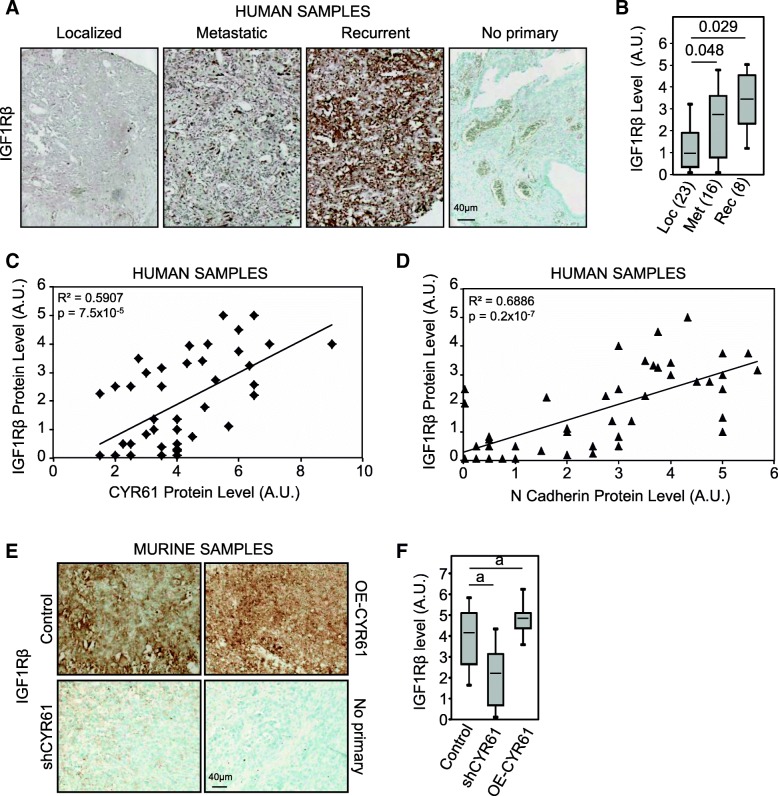
CYR61 controls the expression level of IGF1Rβ. **a** Immunohistochemical staining for IGF1Rβ in human samples derived from localized, metastatic or recurrent osteosarcoma. **b** Box plot of IHC staining score for IGF1Rβ in human samples. a: *p* < 0.05 vs. localized tumor. **c** Spearman correlation between IGF1Rβ expression levels and CYR61 expression levels in human samples. The black line shows the regression line. **d** Spearman correlation between IGF1Rβ expression levels and N-cadherin expression levels in human samples. The black line shows the regression line. **e** Immunohistochemical staining for IGF1Rβ in cell line-derived xenograft of control, CYR61 silenced or CYR61-overexpressing cells. **f** Box plot of IHC staining score for IGF1Rβ in murine samples. a: *p* < 0.05 vs. control cells

In order to determine the upstream molecule triggering the CYR61-dependent signaling pathway leading to EMT-like process, the protein expression level of receptors with tyrosine kinase activity (RTKs) was assessed on TMA by IHC. As previously reported [22], the average IHC staining score for EGFR and PDGFR increased with tumor aggressiveness (Additional file 3: Figure S3A-B). However, neither EGFR nor PDGFRα expression levels correlate with CYR61 protein level (Additional file 3: Figure S3C-D). In addition, neither EGFR nor PDGFRα expression levels correlate with N-cadherin protein levels (Additional file 3: Figure S3E-F). The expression level of IGF1Rβ was higher in metastatic (2.7-fold; *p* = 0.0217) and even higher in recurrent tumor tissues (3.3-fold, *p* = 3 × 10^− 4^) than in localized primary tumor tissues (Fig. 5a-b). A Cox proportional-hazards regression model confirmed that IGF1Rβ up-regulation predicted higher risk of death (nonlinear risk; *p* = 0.026). The IGF1Rβ expression level positively correlates with CYR61 and N-cadherin protein level in tumor tissues (Fig. 5c-d).

The expression level of IGF1Rβ was assessed by IHC in primary tumors generated by injection of CYR61-modified and parental cells (Fig. 5e-f). Tumors derived from CYR61 silenced cells showed a reduced expression of IGF1Rβ as compared to control tumor cells (− 49%, p = 3 × 10^− 5^). Conversely, tumors derived from CYR61 overexpressing cells showed a slight increased expression of IGF1Rβ (+ 20%, *p* = 0.0376).

Altogether, these results indicate that CYR61 expression level associates with IGF1Rβ but not with EGFR or PDGFR expression in osteosarcoma.

### CYR61 controls the expression level of IGF1Rβ

**Fig. 6:**
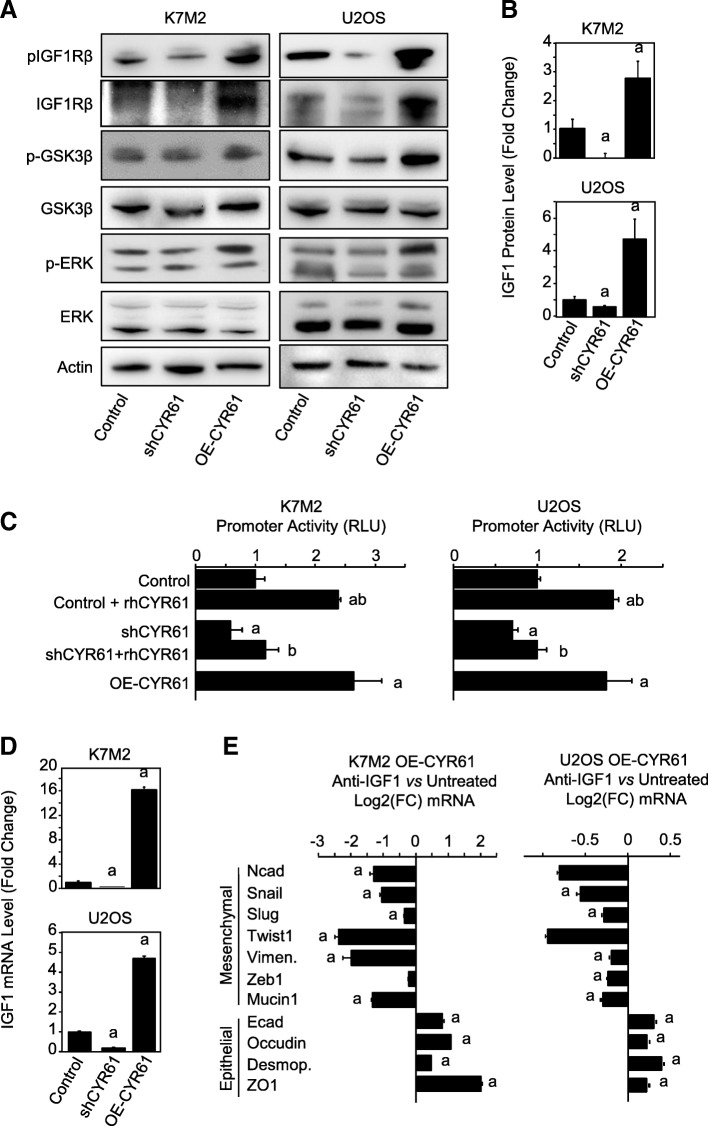
CYR61 controls the expression level of IGF1Rβ and IGF1. **a** Expression pattern of IGF1Rβ protein in K7M2 and U2OS cell lines, as assessed by Western blot. Actin was used as loading control. **b** Expression pattern of IGF1 protein in K7M2 and U2OS cell lines, as assessed by ELISA. Results are expressed as mean ± standard deviation. a: *p* < 0.05 vs. control cells. **c** Relative luciferase activity of IGF1Rβ promoter construct in K7M2 and U2OS cell lines incubated in the presence or absence of recombinant CYR61. a: *p* < 0.05 vs. control cells; b: *p* < 0.05 vs. untreated. **d** Expression pattern of IGF1 in K7M2 and U2OS cell lines, as assessed by RT-qPCR. The relative mRNA levels were calculated using the 2^–ΔΔCT^ method and expressed as Log_2_ of fold change (mean ± standard deviation). a: *p* < 0.05 vs. control cells. **e** Expression pattern of a panel of mesenchymal and epithelial markers in CYR61 overexpressing cell lines, incubated with/without neutralizing anit-IGF antibody, as assessed by RT-qPCR. The relative mRNA levels were calculated using the 2^–ΔΔCT^ method and expressed as Log_2_ of fold change (mean ± standard deviation). a: *p* < 0.05 vs. control cells

In order to characterize the relationship between CYR61 and IGF1Rβ in osteosarcoma cells, control and CYR61-modified osteosarcoma cells were tested for IGF1Rβ expression levels (Fig. 6a). CYR61 silencing led to a reduced expression of IGF1Rβ in both murine K7 M2 and human U2OS cell lines, as assessed by western blot. Conversely, CYR61 overexpression led to an increased expression of IGF1Rβ. The activity of IGF1Rβ, reflected by its phosphorylation levels was correlated to the expression level of the receptor. The phosphorylation state of downstream effectors such as GSK3β was correlated to IGF1Rβ activity and CYR61 levels (Fig. 6a).

Control and CYR61-modified osteosarcoma cells were then tested for IGF1Rβ promoter activity using gene reporter assay (Fig. 6c). CYR61 silencing led to a decrease in relative luciferase activity (− 30 and − 40%, *p* < 0.05) compared to control cells whereas CYR61 overexpression led to a higher luciferase activity in both murine K7 M2 and human U2OS models (1.8- and 2.6-fold, *p* < 0.05). The supplementation of culture medium with recombinant CYR61 also increased relative luciferase activity in control murine K7 M2 and human U2OS cells (2.3-fold and + 90%, respectively, *p* < 0.05), resulting in a relative luciferase activity comparable to the CYR61-overexpressing cells. Similarly, supplementation with recombinant CYR61 increased relative luciferase activity in CYR61-silenced murine K7 M2 and human U2OS cells (1.4- and 2-fold, respectively, *p* < 0.05), resulting in a relative luciferase activity comparable to the control cells.

Altogether, these results indicate that CYR61 increased IGF1Rβ promoter activity, leading to increased IGF1Rβ expression levels in osteosarcoma cells and tumor.

### CYR61 controls the expression level of IGF1 in osteosarcoma cells

Control and CYR61-modified osteosarcoma cells were tested for IGF1 expression levels. CYR61 silencing led to a reduced expression of IGF1 in both murine K7 M2 and human U2OS cell lines, as assessed by real time RT-qPCR (up to − 90%, p < 0.05; Fig. 6d). Conversely, CYR61 overexpression led to an increased expression of IGF1 (16- and 4.7-fold, respectively, *p* < 0.05). Those correlated variations were confirmed at a protein level as assessed by ELISA on conditioned media (Fig. 6b).

CYR61-overexpressing cells were incubated in the presence of neutralizing anti-IGF1 antibody for 24 h before evaluation of mRNA levels of EMT markers (Fig. 6e). All tested mesenchymal markers were down-regulated in the presence of anti-IGF1 antibody, whereas all epithelial markers were up-regulated.

These results indicate that CYR61 induced IGF1 synthesis by osteosarcoma cells, cooperating to the acquisition of a more pronounced mesenchymal phenotype. They also suggest that the inductive effects of the overexpression of CYR61 on EMT markers could be limited by anti-IGF1 neutralizing antibody.

### CYR61 influences cell motility and cell-cell interaction through the IGF1/IGF1Rβ pathway

**Fig. 7:**
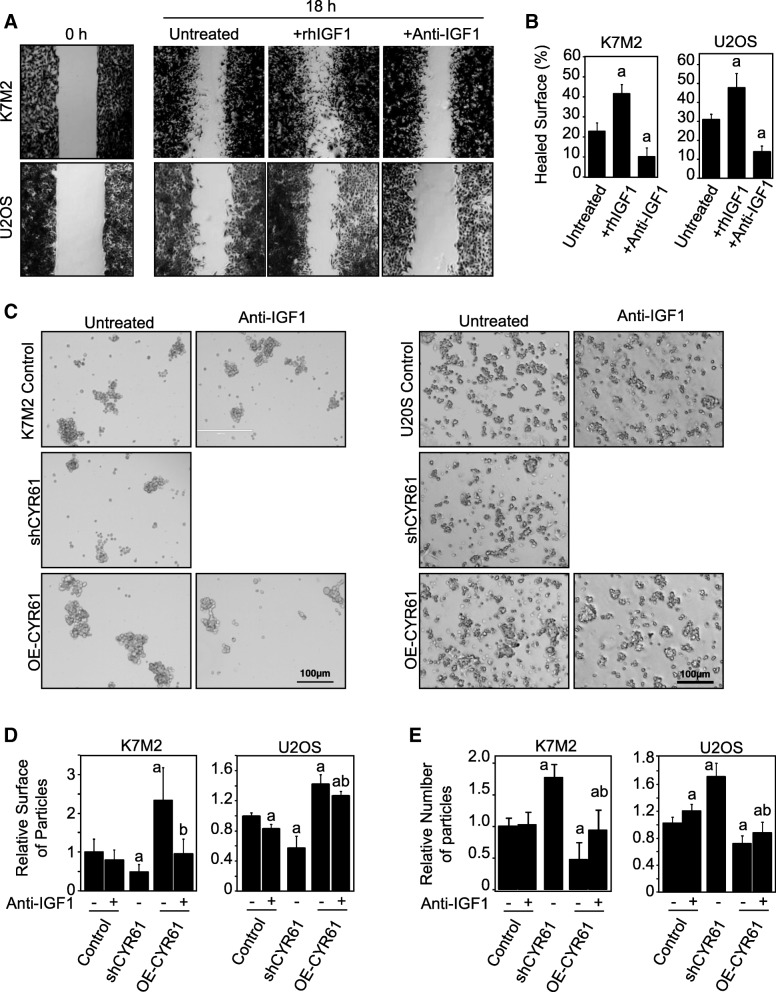
CYR61 influences cell motility and cell-cell interaction through the IGF1/IGF1Rβ pathway. **a** Migration of K7M2 and U2OS cell lines incubated in the presence or absence of recombinant IGF1 or neutralizing anti-IGF1 antibody, as assessed by wound healing assays. Pictures were taken at time 0 and 18 hrs after the wound. **b** Quantitative evaluation of the wound healing. Results are expressed as mean ± standard deviation. a: *p* < 0.05 vs. control cells. **c** Cell aggregation assay of K7M2 and U2OS cell lines cultured in the presence or absence of neutralizing anti-IGF1 antibody. **d** Quantitative evaluation of the relative aggregate surface. Results are expressed as mean ± standard deviation. a: *p* < 0.05 vs. control cells; b: *p* < 0.05 vs. untreated. e Quantitative evaluation of the relative number of particles. Results are expressed as mean ± standard deviation. a: *p* < 0.05 vs. control cells; b: *p* < 0.05 vs. untreated

In order to evaluate the involvement of IGF1/IGF1Rβ pathway in the pro-metastatic effect of CYR61, cells were tested for cell migration and aggregation under different culture conditions. As expected, culture medium supplementation with recombinant IGF1 led to a significant increase in in vitro cell migration capacities for both K7 M2 and U2OS cells (+ 81% and + 53%, respectively; *p* < 0.05; Fig. 7a-b). On the other hand, the addition of a blocking/neutralizing anti-IGF1 antibody led to an inhibition of cell migration capacities (− 52% and − 43%, respectively; *p* < 0.05).

K7 M2 and U2OS osteosarcoma cells were then tested for anchorage-independent cell aggregation (Fig. 7c-e). CYR61-silenced cells were less able to interact with one another and form smaller aggregates than control cells. The silencing of CYR61 thus led to a decrease in the surface of aggregates (− 51%, *p* < 0.05), and increase in the aggregate number (+ 76%, *p* < 0.05). Conversely, CYR61-overexpressing cells can more easily adhere to each other and form larger cell aggregations (2.3-fold and + 46%, respectively, p < 0.05), and less numerous aggregates (− 53%, p < 0.05). Supplementation of culture medium with a blocking/neutralizing anti-IGF1 antibody normalized the surface and number of aggregates formed from CYR61-overexpressing cells.

These results suggest that CYR61 controls osteosarcoma cell-cell interactions and cell motility through an IGF-dependent process.

### CYR61 level influences the expression of EMT markers and cell motility through a JNK-dependent pathway

**Fig. 8:**
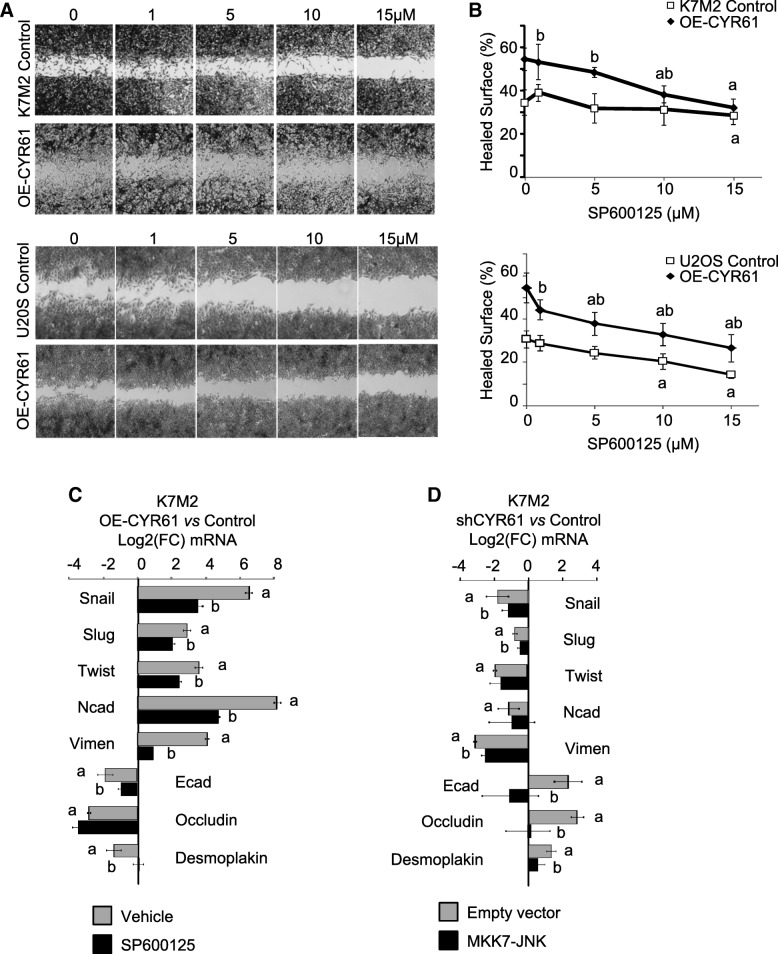
CYR61 influences cell motility and the expression of EMT markers through a JNK-dependent pathway. **a** Migration of K7 M2 and U2OS cell lines incubated in the presence or absence of the JNK inhibitor SP600125 (15 µM), as assessed by wound healing assays. Pictures were taken at time 0 and 18 h after the wound. **b** Quantitative evaluation of the wound healing. Results are expressed as mean ± standard deviation. a: *p* < 0.05 vs. control cells; b: *p* < 0.05 vs. untreated. **c** Expression pattern of a panel of mesenchymal and epithelial markers in K7M2 cell lines cultured in the presence or absence of the JNK inhibitor SP600125, as assessed by RT-qPCR. The relative mRNA levels were calculated using the 2^–ΔΔCT^ method and expressed as Log_2_ of fold change (mean ± standard deviation). a: *p *< 0.05 vs. control cells; b: *p* < 0.05 vs. untreated. **d** Expression pattern of a panel of mesenchymal and epithelial markers in K7M2 cell lines transfected with a MKK7-JNK expressing vector or the corresponding empty vector, as assessed by RT-qPCR. The relative mRNA levels were calculated using the 2^–ΔΔCT^ method and expressed as Log_2_ of fold change (mean ± standard deviation). a: *p* < 0.05 vs. control cells; b: *p* < 0.05 vs. untreated

As we previously demonstrated that osteosarcoma cell motility and invasiveness are dependent on JNK pathway [23], CYR61-modified cells were tested for in vitro cell migration in the presence of the JNK inhibitor SP600125. Concentrations as high as 10–15 μM were required to significantly reduce JNK phosphorylation levels (Additional file 4: Figure S4). Those high concentrations led to a detectable reduction of wound healing capacities for K7 M2 and U2OS control cells (Fig. 8a-b). In contrast, low concentrations of the JNK inhibitor SP600125 allowed significantly affecting CYR61-overexpressing K7 M2 and U2OS cells. Supplementation with 10–15 μM JNK inhibitor led to a complete abrogation of the stimulatory effect of CYR61 on wound healing.

The gene expression level of EMT markers was assessed by real-time RT-qPCR. Medium supplementation with JNK inhibitor SP600125 (15 μM) led to reduced expression levels of mesenchymal-related markers (Snail-1, Snail-2/Slug, Twist1, N-cadherin, Vimentin) and enhanced expression levels of epithelial-related markers (E-cadherin, Occludin, Desmoplakin) in CYR61-overexpressing cells (Fig. 8c). On the other hand, transient transfection of CYR61-silenced cells to express a constitutively active form of MKK7 coupled to JNK led to an enhanced expression of mesenchymal-related markers, and to a reduction in gene expression levels of epithelial-related markers (Fig. 8d).

Altogether, these results indicate that CYR61 modulates EMT markers expression and cell motility at least in part through modulation of JNK activity.

## Discussion

Our results provide evidence that in osteosarcoma cells CYR61 plays a key role in the modulation of expression of some phenotypic markers leading to a process similar to the epithelial-to-mesenchymal transition (EMT) observed in tumors of epithelial origin. This resulted in the promotion of cell motility and invasiveness, leading to the enhancement of metastatic dissemination of the primary tumor. Our results also demonstrate that a major effector pathway involved was the modulation of IGF1 and its receptor IGF1Rβ by CYR61. The silencing of CYR61 or the blockade of IGF1/IGF1Rβ pathway significantly reduced the pro-metastatic activity of osteosarcoma cells, as demonstrated in vitro or in preclinical models.

The involvement of a mesenchymal transition process in the onset and progression of sarcomas of different origins has been very recently discussed in the review of Kahlert and colleagues [24], suggesting EMT and mesenchymal-to-epithelial transition (MET)-related processes in non-epithelial tumors. Osteosarcoma cells have been reported to undergo an “EMT-like” process that associate with their metastatic ability [17–20]. We found more appropriate to use the term of “EMT-like” to describe the phenotype observed in our models. Our data indeed demonstrate that CYR61 overexpression led to (i) a reduction in expression level of molecular markers considered as epithelial such as E-cadherin, occludin, zonula occludens-1 or desmoplakin, leading to decreased cell aggregation; (ii) upregulation of mesenchymal molecular markers such as N-cadherin, vimentin, or mucin-1; (iii) cytoplasmic elongation and acquisition of a more fibroblastic shape; and,, (iv) increased migratory and invasive potential, and also (v) enhanced resistance to basal cell death and chemotherapy compounds, as we previously demonstrated [11, 12]. Conversely, silencing CYR61 in osteosarcoma cells led to the reverse process, namely MET where cells adopted a more cuboid shape, tended to aggregate because of higher level of cell-cell adhesion proteins (shift from N-cadherin to E-cadherin), and secreted less MMPs. Altogether, our results clearly point to CYR61 as a critical player in osteosarcoma behavior by affecting both tumor cells (cytoskeleton reorganization, cell-cell contacts…) and microenvironment of primary tumor (extracellular matrix synthesis, proteases expression, neo-angiogenesis).

Our results support the proposal of the induction of EMT-like in osteosarcoma cells when CYR61 expression rises. Those variations in general hallmarks of EMT were also detected in vivo in a preclinical murine model of tumors derived from genetically modified osteosarcoma cells expressing various controlled levels of CYR61. We further confirmed a positive correlation between tumor grade, CYR61, and N-cadherin expression levels in tissue samples originating from patients suffering of localized and metastatic osteosarcoma. Our results are consistent with the reported induction of EMT under overexpression of CYR61 in laryngeal squamous cell carcinoma, pancreatic cancer cell lines, gastric cancer, osteosarcoma or chondrosarcoma [25–29]. EMT being a key process for tumor cells to disseminate to distant organs, our results provide further evidence that CYR61 is a key inducer of osteosarcoma metastatic spreading.

From a mechanistic point of view, members of CCN family can interfere with signal transduction of growth factors such as FGF, PDGF, TGFβ or BMPs [30, 31]. Recently, insulin-like growth factor 2 receptor (IGF2R) has been suggested as CTGF-interacting protein in fibroblasts [32]. With regard to CYR61, integrin receptors and heparin-sulfate proteoglycans (HSPGs) binding sites have been largely documented [30]. Among its four conserved structural domains, CYR61 includes an insulin-like growth factor-binding protein (IGFBP) homology domain a reason why it was formerly called IGFBP10. IGFs are the most abundant growth factors produced by osteoblasts [33] and stored in bone matrix. IGF increases bone formation by regulating the proliferation, differentiation, and apoptosis of osteoblasts by binding IGF receptor type 1 (IGF1Rβ). This IGF system plays an important role in the development of osteosarcoma [34]. In this study, we showed that IGF1Rβ expression levels increases with tumor aggressiveness status in our preclinical murine model and in human tumor samples. This is in accordance with the correlation mentioned between elevated IGF1R mRNA expression and distant metastasis occurrence in human osteosarcoma tumor [35, 36].

We also demonstrated that CYR61 levels influence IGF1 and IGF1Rβ expression (at a transcriptional level), resulting in the modulation of some IGF1Rβ downstream signaling such as JNK. Activating JNK-MAPKs signaling pathway promotes EMT in tumor cells (reviewed in [37]). Our results conformed well to that since the inhibition or activation of JNK activity interferes with the relative expression levels of epithelial and mesenchymal markers in osteosarcoma cells. We also previously demonstrated that, by controlling MMP2/MMP9 expression and activity, the JNK/c-Jun signaling pathway plays a key role in osteosarcoma potential for metastasis [23, 38]. In the present study, we finally established that CYR61 influences the expression of EMT markers and cell motility through a JNK-dependent pathway.

Much effort has been devoted to develop anticancer agents that block IGF/IGFR signaling pathway. In vitro and preclinical assays evaluating inhibitors or antagonists of IGF receptors or ligands have reported positive effects in regulating tumorigenic and metastatic properties of osteosarcoma cells [39–43]. Those promising results encouraged the assessment of targeting IGF pathway in patients. Phase-I and phase-II clinical trials evaluating IGF-1R antibodies as therapy for osteosarcoma patients have returned mixed results. Despite the very small percentage of osteosarcoma patients included in those trials, and thus delicate interpretation of the results, several stable disease and partial/complete response were reported [44–46]. Some clinical trials focusing on monoclonal anti-IGF1R in patients with relapsed and/or recurrent osteosarcoma are ongoing. Revealing the upstream inducer function of CYR61, alias IGFBP10, our results firstly reinforce the importance of the IGF pathway into osteosarcoma pathogenesis, and also open up new therapeutic opportunities for aggressive osteosarcoma or primary tumors exhibiting a high metastatic risk.

Receptors with tyrosine kinase activity (RTKs) are reported substantially involved in development and progression of osteosarcoma [47]. We indeed previously reported that metastatic and recurrent osteosarcoma tumors expressed higher EGFR and PDGFRα levels than localized tumors [22]. The present study provides evidence of a positive correlation between IGF1Rβ expression level and aggressive tumor development. Surprisingly, among the tested RTKs, only IGF1Rβ level variations correlated with that of CYR61, reinforcing the key role of IGF pathway in metastatic osteosarcoma. This also confirms the relevance for developing strategy to block these CYR61-dependent pro-metastatic signals.

As CYR61 need partners to transduce its pro-metastatic signal, the involvement of major signaling effectors should not be overlooked. As an example, the YAP1 (Yes-associated protein)/TAZ (transcriptional coactivator with PDZ-binding motif) are reported to play an important role in tumor initiation and cancer progression, and may be proposed as prognostic biomarker in gastric, breast, ovarian, renal and prostate cancers (reviewed in [48]). The up-regulation of YAP in osteosarcoma tissue has been reported [49, 50]. Cyr61 is one of the transcriptional targets and downstream effectors of YAP/TAZ [51]. It could be speculate that a cooperation or relationship between YAP/TAZ pathway and CYR61 may play a key role in osteosarcoma progression. In a similar way, interference with Wnt/β catenin signaling pathways may be considered as CYR61 is a downstream effector of these substantial contributors to osteosarcoma tumorigenesis [52, 53].

## Conclusions

Our current results provide evidence that CYR61 plays a crucial role in the complex processes of primary tumor dissemination. CYR61 controls the IGF/IGFR levels and triggers an EMT-like process. This allows tumor cells to acquire morphological changes, to increase invasiveness to the surrounding stroma. Combined to our previous studies, reporting the essential promoting role of CYR61 in primary tumor vascularization, this study suggests CYR61 as a novel candidate biomarker of osteosarcoma associated with aggressiveness and with metastasis. Targeting of this molecule could additionally represent a valuable therapeutic strategy in osteosarcoma to prevent cancer progression and metastatic disease. Our data also strongly support the need to evaluate IGF/IGFR pathway under CYR61 context in osteosarcoma patients.

## Methods

### Reagents and antibodies

Recombinant CYR61 and SP600125 were purchased from Sigma-Aldrich (Lyon, France), recombinant IGF-1 was purchased from R&D Systems (Lille, France).

Neutralizing anti-IGF1 antibody was purchased from R&D Systems. Rabbit polyclonal anti-CYR61 and anti-pIGF1Rβ were purchased from Abcam (Cambridge, UK), anti-Actin was purchased from Sigma Aldrich and anti-IGF1Rβ, anti-pGSK3β, anti-pJNK, anti-JNK were purchased from Cell Signaling Technology (Saint Quentin en Yvelines, France). Mouse monoclonal anti-E-cadherin was purchased from Santa Cruz Biotechnologies (Santa Cruz, CA, USA), anti- GSK3β was purchased from Cell Signaling Technology, and anti-IGF1 was purchased from R&D Systems.

### Cell lines and culture

Murine K7 M2 and human U2OS osteosarcoma cells (American Type Culture Collection, Rockville, MD, USA) were transduced with lentiviral vectors encoding either the full-length coding sequence or specific shRNA sequences as previously described [12] to stably increase or reduce CYR61 expression, respectively. All cell lines were cultured in high glucose Dulbecco’s Modified Eagles Medium (DMEM; Invitrogen Corporation, Paisley, Scotland) supplemented with 10% heat inactivated fetal calf serum at 37 °C in an atmosphere of > 95% humidity and 5% CO_2_. Culture media were changed three times a week and regularly tested to ensure absence of mycoplasma.

### Spheroid formation

Osteo-spheroids were generated by seeding 5000 cells/well in poly-Hema coated 96-well round-bottomed plates. Medium was supplemented with Matrigel (4 mg/ml) at day 2 to allow invasion. Images were captured using EVOS Cell Imaging System (Thermo Fisher Scientific).

### RNA extraction

Total RNAs were isolated using TRIzol Reagent (Thermo Fisher Scientific, Courtabœuf, France), according to the manufacturer’s protocol, suspended in H_2_O supplemented with RNAsecure reagent (Thermo Fisher Scientific) and stored at − 80 °C. RNA Integrity was checked using an Agilent 2100 BioAnalyzer to select samples exhibiting RNA Integrity Number > 8.

### Real-time RT-PCR

Total RNA (3 μg) were denatured for 10 min at 70 °C then reverse transcribed at 37 °C for 90 min using 300 U MMLV reverse transcriptase, 15 μg random hexamers, 1 mM deoxynucleoside triphosphate (dNTP) in 30 μl total volume. Real-time quantitative Polymerase Chain Reactions (qPCR) were performed on ViiA7 apparatus (Thermo Fisher Scientific) using SYBrGreen Master kit (Thermo Fisher Scientific) supplemented with 0.5 μM of specific primers (Additional file 5: Table S1 and Additional file 6: Table S2). Thermal conditions were: 15 min at 95 °C for activation then 50 cycles of denaturation at 95 °C for 20 s, 58 °C annealing for 15 s and 72 °C extension for 15 s. Melting curve analysis was included to assure that only one PCR product was formed. The relative amounts of RNA were calculated by the 2^-∆∆Ct^ method.

### Enzyme-linked immunosorbent assays (ELISA)

Conditioned media were collected after 48 h of culture under serum-free conditions and cleared from cell debris by centrifugation. ELISAs were performed immediately for IGF-1, in accordance with the manufacturers’ recommendations (R&D Systems).

### In vitro wound healing

Cells were seeded in each chamber of the Culture-Insert (Ibidi; Martinsried, Germany) and incubated for 24 h in medium supplemented with 2.5% FCS. Confluent cell monolayers were carefully washed once with phosphate buffered saline (PBS), cultured for 18 h, fixed in 75% ethanol and stained with crystal violet (0.05% in ethanol). Recovery of the denuded area was computerized using EVOS digital microscope (Delta Microscopies, Ayguesvives, France). Lesion area surface at time zero was used as matrix for cell number evaluation in other lesion areas. Six replicates were used for each condition and experiments were repeated three times.

### Cell aggregation assay

Cells were seeded (10^5^ cells/cm^2^) in bacteriological grade culture plates. After 24 h, plates were placed on a gyratory shaker for 60 min at 37 °C to allow cell aggregation. Single cells and cell clusters were counted and cell aggregation was evaluated using ImageJ software. Experiments were repeated three times with 3–4 replicates for each condition.

### Cell adhesion assay

Wells were coated or not with type I collagen (5 μg/mL), laminin (0.5 μg/mL) or fibronectin (2 μg/mL) by overnight incubation at 37 °C in 5% CO_2_. The coated wells were washed twice with PBS followed immediately by cell seeding (10^5^ cells/cm^2^). After 30 min, the supernatant was removed, cell layers were carefully washed once with PBS and fixed with 4% paraformaldehyde for 20 min at + 4 °C. Wells were stained with crystal violet (0.05% in ethanol) before computerization using EVOS digital microscope.

### Western blot

Cell lysates were prepared as previously described [54]. Proteins (30 μg) were resolved on 12% SDS-PAGE and electro-transferred onto PVDF membranes. Those membranes were incubated for 2 h in blocking buffer (Sigma Aldrich), then overnight at 4 °C with specific primary antibodies (0.5 μg/mL). Membranes were washed twice with [50 mM Tris/HCl pH 7.4, 150 mM NaCl, 0.1% (*v*/v) Tween-20] (TBST) and incubated for 2 h with appropriate HRP-conjugated secondary antibody (1/20,000). After final washes, the signals were visualized with enhanced chemiluminescence western blotting detection reagent (Thermo Fisher Scientific) on the ChemiDoc XRS+ apparatus (BioRad Laboratories, Marnes-la-Coquette, France) and quantified using the ImageJ software.

### Reporter assay

Cells were seeded (4000 cells/cm^2^) in 6-well plates and transiently co-transfected the day after with 0.2 μg/well pOLUC-IGF1R [55] and 0.2 ng/well of pCH110 (Addgene, Cambridge, MA, USA) using Lipofectamin2000 reagent (Thermo Fisher Scientific) according to manufacturer’s recommendations. Cells were cultured for further 36 h. Transcriptional activity was evaluated using a Luciferase Reporter Assay System according to the manufacturer’s protocol (Promega) and corrected to beta-galactosidase activity evaluated using β-Gal reporter gene assay (Roche, Meylan, France).

### Cell-line-derived xenograft (CDX) models

In vivo assay was performed as previously described [11, 12]. Briefly, after acclimatization for 7 days, 5-week old BALB/c mice (Charles River, Arbresle, France) were intramuscularly injected with K7 M2 osteosarcoma cells (10^6^ cells/15 μl PBS) in both thigh muscles under isoflurane/air inhalational anaesthesia. At day 28 after cell injection, mice were killed by CO_2_ asphyxiation. Muscle infiltrated with tumor tissues and lungs were collected, formalin-fixed then embedded in paraffin.

### Osteosarcoma tissue microarray (TMA)

An osteosarcoma tissue microarray composed of 233 tissue cores included in paraffin (205 tumor samples and 28 normal bone samples) was used for an IHC study. The characteristics of the patients are summarized in Additional file 1: Figure S1A-E.

### Hematoxylin and eosin (H&E) staining and immunohistochemistry

The deparaffinization of formalin-fixed paraffin-embedded tissue sections (5 μm) was performed in xylene before the dehydration through a graded alcohol series. Lung and tumor tissue samples were stained with hematoxylin and eosin (H&E) for histological analyses. Tumor tissue sections were micro-waved in citrate buffer (pH 6.0) for epitopes unmasking then incubated for 5 min in 3% H_2_O_2_ for endogenous peroxidase quenching. The sections were incubated with anti-CYR61 (5 μg/mL), anti-N-Cadherin (20 μg/mL, Abcam), anti-E-Cadherin (4 μg/mL) or anti-IGF1Rβ (2 μg/mL) antibodies overnight at 4 °C, in humidified atmosphere. Primary antibodies were detected using Vectastain Elite ABC system (Vector Laboratories Ltd., Peterborough, UK). Negative control sections were incubated with antibody diluent, without primary antibody, followed by incubation with secondary antibody. Signal intensity was quantified on 15–18 fields located outside of the necrotic areas and without the remaining muscular fibers using ImageJ 1.51p. The TMA slides were processed as above. Signal intensity was estimated by two observers without prior information about TMA spots and thereafter related to clinical information.

### Statistical analysis

Comparisons between data were performed using the two-factor analysis of variance using the statistical package ANOVA. Preclinical data was analyzed using non-parametric Mann-Whitney test. A minimal level of *P* < 0.05 was considered statistically significant.

## Additional files

Additional file 1: **Figure S1.** Characteristics of patients whose tumor samples are included in tissue micro-array (TMA). A total of 231 core samples were collected from 37 patients with osteosarcoma (205 tumor samples), and 28 normal bone samples. (A) Frequency distribution of patients by age (years) at diagnosis. (B) Histologic subtypes (%). (C) Kaplan-Meier survival curve for overall survival of Male and Female. (D) Kaplan-Meier survival curve for overall survival of patients with localized of metastatic tumor at diagnosis. (E) Kaplan-Meier survival curve for overall survival of patients experiencing relapse or not. (PPTX 93 kb)

Additional file 2: **Figure S2.** CYR61 controls osteosarcoma cell phenotype in vitro. (A) Morphology of K7 M2 and U2OS osteosarcoma control, CYR61 silenced and CYR61 overexpressing cells, grown in medium supplemented with 10% Fetal Calf Serum. (B) Correlation between the relative maximal cell length of K7 M2 and U2OS cells. Results are expressed as mean ± standard deviation (*n* > 2000 cells/field; at least 8 field/condition; experiments repeated twice). (C) Brightfield imaging of osteo- spheroids. (D) Quantitative evaluation of the relative aggregate surface. Results are expressed as mean ± standard deviation (*n* = 6). a: *p* < 0.05 vs. control cells. (E) Relative K7 M2 cell number adherent to the indicated surface, after 30 min incubation. Results are expressed as Log_2_ of fold change (mean ± standard deviation). a: p < 0.05 vs. control cells. (PPTX 3906 kb)

Additional file 3: **Figure S3.** CYR61 expression level does not correlate with those of EGFR or PDGFR. Box plot of IHC staining scores for EGFR (A), and PDGFR (B). Spearman correlation between EGFR (C) and PDGFR (D) expression levels and CYR61 expression levels in human samples. Spearman correlation between EGFR (E) and PDGFR (F) expression levels and N-cadherin expression levels in human samples. (PPTX 201 kb)

Additional file 4: **Figure S4.** SP600125 inhibitory effect on JNK phosphorylation. Expression pattern of phospho-JNK and phospho-ERK1/2 as well as corresponding total protein in K7 M2 and U2OS cell lines, cultured ON in the presence of increasing concentration of SP600125, as assessed by Western blot. Actin was used as loading control. (PPTX 2635 kb)

Additional file 5: **Table S1.** Primer sequences for real time quantitative PCR (mouse). (DOCX 16 kb)

Additional file 6: **Table S2.** Primer sequences for real time quantitative PCR (human). (DOCX 16 kb)

## Abbreviations

BMP: Bone morphogenetic protein
CTGF: Connective tissue growth factor
CYR61: Cysteine rich protein 61
EFS: Event free survival
EGFR: Epidermal growth factor receptor
EMT: Epithelial-to-mesenchymal transition
ERK-MAPKs: Extracellular signal related kinases-mitogen activated protein kinases
FFPE: Formaldehyde fixed paraffin embedded
FGFR: Fibroblast growth factor receptor
GSK3: Glycogen synthase kinase 3
HGF: Hepatocyte growth factor
HSPG: Heparan sulfate proteoglycan
IGF1Rβ: Insulin-like growth factor 1 receptor beta
IGFBP: Insulin-like growth factor binding protein
IHC: Immunohistochemistry
JNK: c-Jun N-terminal kinases
MET: Mesenchymal-to-epithelial transition
MKK7: Mitogen activated protein kinase kinase 7
MMP: Matrix metalloproteinase
NOV: Nephroblastoma overexpressed
PDGFR: Platelet derived growth factor receptor
RTK: Receptor with tyrosine kinase activity
TGFβ: Transforming growth factor beta
TIMP: Tissue inhibitor of metalloproteinases
TMA: Tissue micro array
WISP: WNT1-inducible-signaling pathway
