# Supplementary material for: CYR61 triggers osteosarcoma metastatic spreading via an IGF1Rβ-dependent EMT-like process
Source: BMC Cancer. 2019 Jan 14;19:62. doi: 10.1186/s12885-019-5282-4 (PMC6332662; doi:10.1186/s12885-019-5282-4)
Supplement: Supplementary file 1 — Figure S1. Characteristics of patients whose tumor samples are included in tissue micro-array (TMA). A total of 231 core samples were collected from 37 patients with osteosarcoma (205 tumor samples), and 28 normal bone samples. (A) Frequency distribution of patients by age (years) at diagnosis. (B) Histologic subtypes (%). (C) Kaplan-Meier survival curve for overall survival of Male and Female. (D) Kaplan-Meier survival curve for overall survival of patients with localized of metastatic tumor at diagnosis. (E) Kaplan-Meier survival curve for overall survival of patients experiencing relapse or not. (PPTX 93 kb) [file 12885_2019_5282_MOESM1_ESM.pptx]

## Slide 1
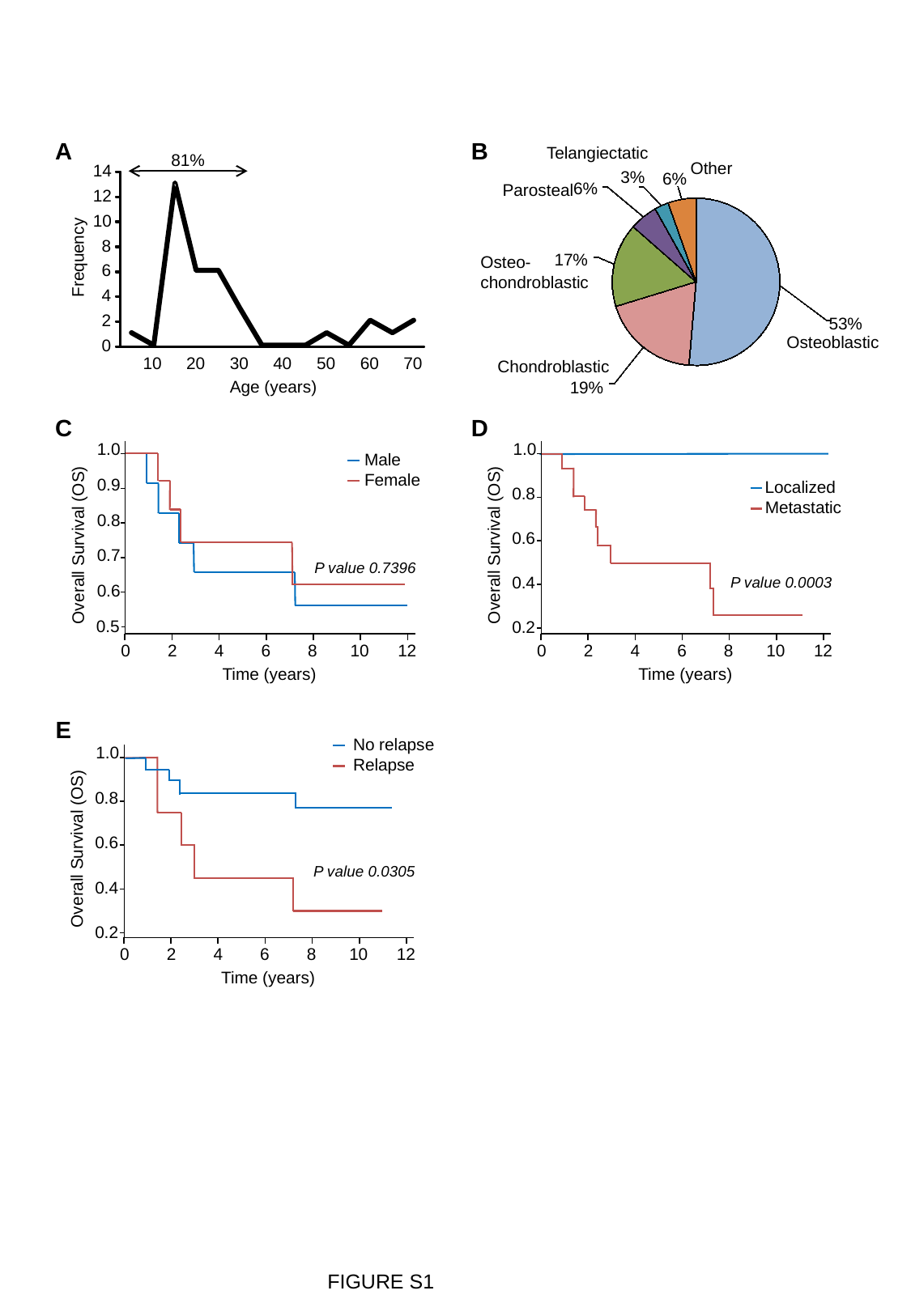

A
B
Telangiectatic
Other
3%
6%
6%
Parosteal
17%
Osteo-
chondroblastic
53%
Osteoblastic
Chondroblastic
19%
81%
14
12
10
8
Frequency
6
4
2
0
10
20
30
40
50
60
70
Age (years)
C
D
1.0
Male
Female
0.9
0.8
Overall Survival (OS)
0.7
P value 0.7396
0.6
0.5
0
2
4
6
8
10
12
Time (years)
1.0
Localized
0.8
Metastatic
0.6
Overall Survival (OS)
0.4
P value 0.0003
0.2
0
2
4
6
8
10
12
Time (years)
E
No relapse
1.0
Relapse
0.8
0.6
Overall Survival (OS)
P value 0.0305
0.4
0.2
0
2
4
6
8
10
12
Time (years)
FIGURE S1
