# Supplementary material for: CYR61 triggers osteosarcoma metastatic spreading via an IGF1Rβ-dependent EMT-like process
Source: BMC Cancer. 2019 Jan 14;19:62. doi: 10.1186/s12885-019-5282-4 (PMC6332662; doi:10.1186/s12885-019-5282-4)
Supplement: Supplementary file 2 — Figure S2. CYR61 controls osteosarcoma cell phenotype in vitro. (A) Morphology of K7 M2 and U2OS osteosarcoma control, CYR61 silenced and CYR61 overexpressing cells, grown in medium supplemented with 10% Fetal Calf Serum. (B) Correlation between the relative maximal cell length of K7 M2 and U2OS cells. Results are expressed as mean ± standard deviation (n > 2000 cells/field; at least 8 field/condition; experiments repeated twice). (C) Brightfield imaging of osteo- spheroids. (D) Quantitative evaluation of the relative aggregate surface. Results are expressed as mean ± standard deviation (n = 6). a: p < 0.05 vs. control cells. (E) Relative K7 M2 cell number adherent to the indicated surface, after 30 min incubation. Results are expressed as Log2 of fold change (mean ± standard deviation). a: p < 0.05 vs. control cells. (PPTX 3906 kb) [file 12885_2019_5282_MOESM2_ESM.pptx]

## Slide 1
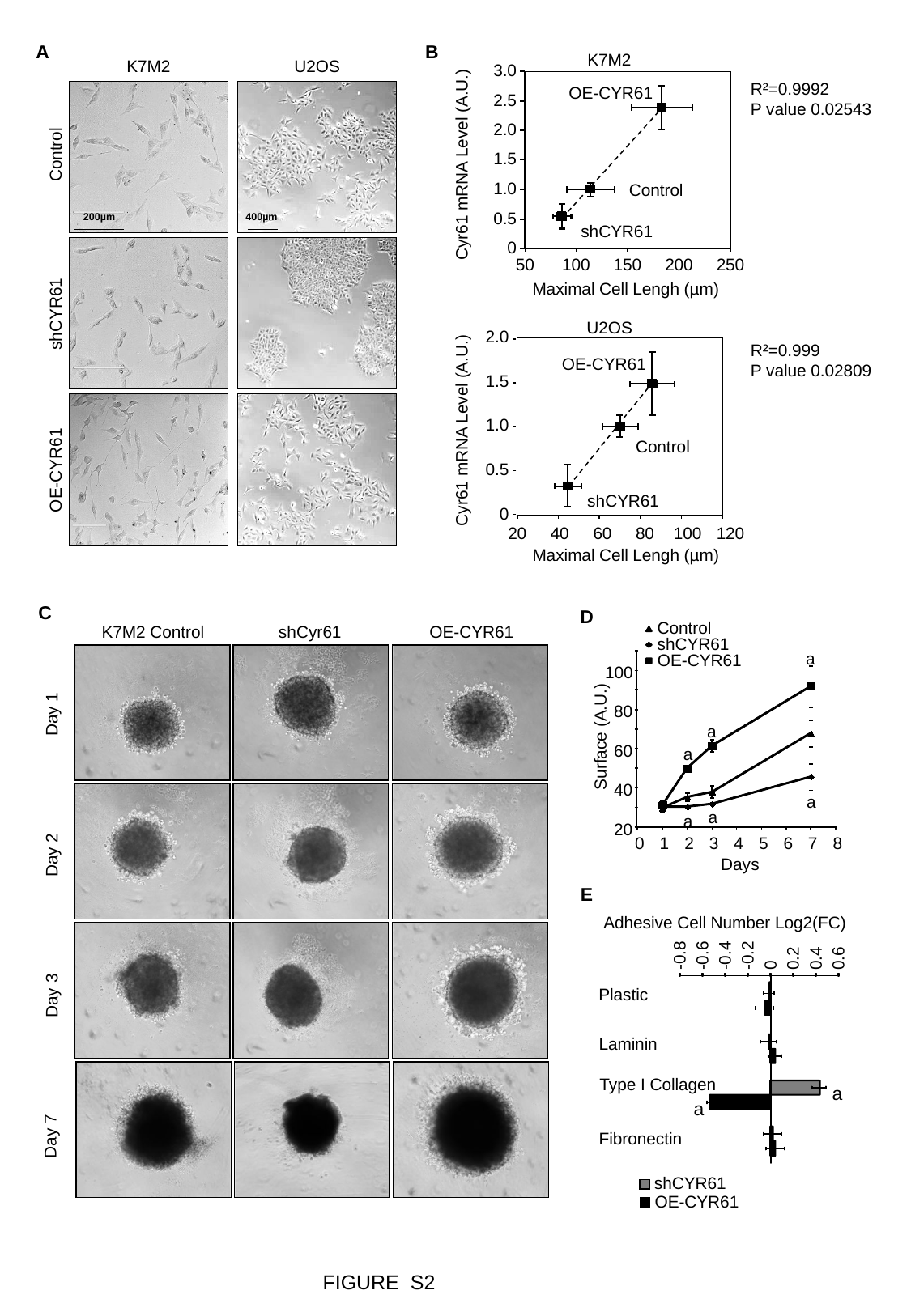

A
B
K7M2
3.0
Cyr61 mRNA Level (A.U.)
R²=0.9992
P value 0.02543
OE-CYR61
2.5
2.0
1.5
Control
1.0
0.5
shCYR61
0
50
100
150
200
250
Maximal Cell Lengh (µm)
U2OS
2.0
Cyr61 mRNA Level (A.U.)
R²=0.999
P value 0.02809
OE-CYR61
1.5
1.0
Control
0.5
shCYR61
0
20
40
60
80
100
120
Maximal Cell Lengh (µm)
K7M2
U2OS
Control
100µm
200µm
400µm
shCYR61
OE-CYR61
C
D
K7M2 Control
shCyr61
OE-CYR61
Day 1
Day 2
Day 3
Day 7
Control
shCYR61
a
OE-CYR61
100
80
a
Surface (A.U.)
a
60
40
a
a
a
20
0
1
2
3
4
5
6
7
8
Days
E
Adhesive Cell Number Log2(FC)
-0.8
-0.6
-0.4
-0.2
0.2
0.4
0.6
0
Plastic
Laminin
Type I Collagen
a
a
Fibronectin
shCYR61
OE-CYR61
FIGURE S2
