# Supplementary material for: CYR61 triggers osteosarcoma metastatic spreading via an IGF1Rβ-dependent EMT-like process
Source: BMC Cancer. 2019 Jan 14;19:62. doi: 10.1186/s12885-019-5282-4 (PMC6332662; doi:10.1186/s12885-019-5282-4)
Supplement: Supplementary file 3 — Figure S3. CYR61 expression level does not correlate with those of EGFR or PDGFR. Box plot of IHC staining scores for EGFR (A), and PDGFR (B). Spearman correlation between EGFR (C) and PDGFR (D) expression levels and CYR61 expression levels in human samples. Spearman correlation between EGFR (E) and PDGFR (F) expression levels and N-cadherin expression levels in human samples. (PPTX 201 kb) [file 12885_2019_5282_MOESM3_ESM.pptx]

## Slide 1
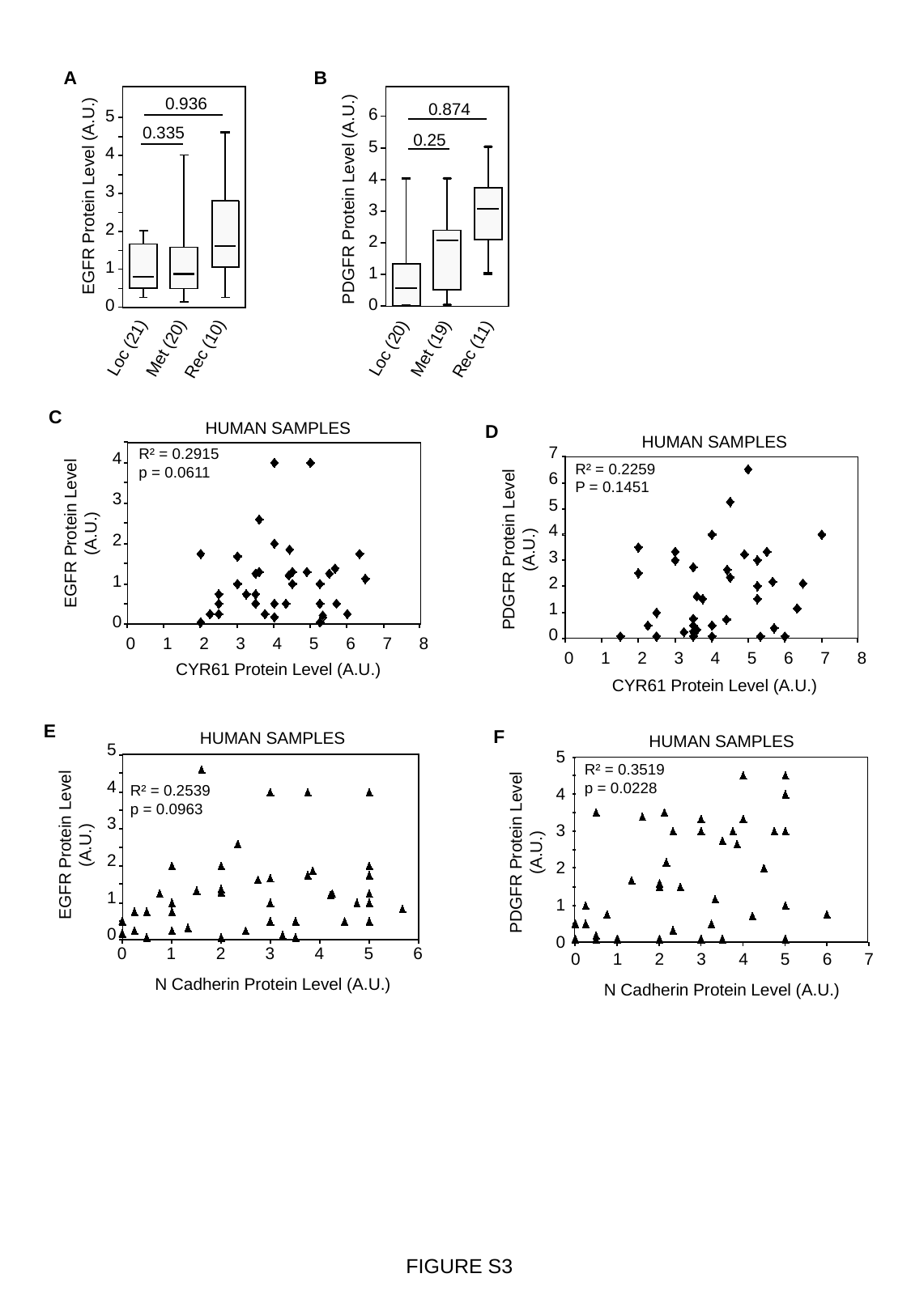

A
0.936
5
0.335
4
3
EGFR Protein Level (A.U.)
2
1
0
Loc (21)
Met (20)
Rec (10)
B
0.874
6
0.25
5
4
PDGFR Protein Level (A.U.)
3
2
1
0
Loc (20)
Met (19)
Rec (11)
C
HUMAN SAMPLES
R² = 0.2915
p = 0.0611
4
3
EGFR Protein Level
(A.U.)
2
1
0
0
1
2
3
4
5
6
7
8
CYR61 Protein Level (A.U.)
D
HUMAN SAMPLES
7
R² = 0.2259
P = 0.1451
6
5
4
PDGFR Protein Level
(A.U.)
3
2
1
0
0
1
2
3
4
5
6
7
8
CYR61 Protein Level (A.U.)
E
HUMAN SAMPLES
5
4
R² = 0.2539
p = 0.0963
3
EGFR Protein Level
(A.U.)
2
1
0
0
1
2
3
4
5
6
N Cadherin Protein Level (A.U.)
F
HUMAN SAMPLES
5
R² = 0.3519
p = 0.0228
4
3
PDGFR Protein Level
(A.U.)
2
1
0
0
1
2
3
4
5
6
7
N Cadherin Protein Level (A.U.)
FIGURE S3
