# Supplementary material for: CYR61 triggers osteosarcoma metastatic spreading via an IGF1Rβ-dependent EMT-like process
Source: BMC Cancer. 2019 Jan 14;19:62. doi: 10.1186/s12885-019-5282-4 (PMC6332662; doi:10.1186/s12885-019-5282-4)
Supplement: Supplementary file 4 — Figure S4. SP600125 inhibitory effect on JNK phosphorylation. Expression pattern of phospho-JNK and phospho-ERK1/2 as well as corresponding total protein in K7 M2 and U2OS cell lines, cultured ON in the presence of increasing concentration of SP600125, as assessed by Western blot. Actin was used as loading control. (PPTX 2635 kb) [file 12885_2019_5282_MOESM4_ESM.pptx]

## Slide 1
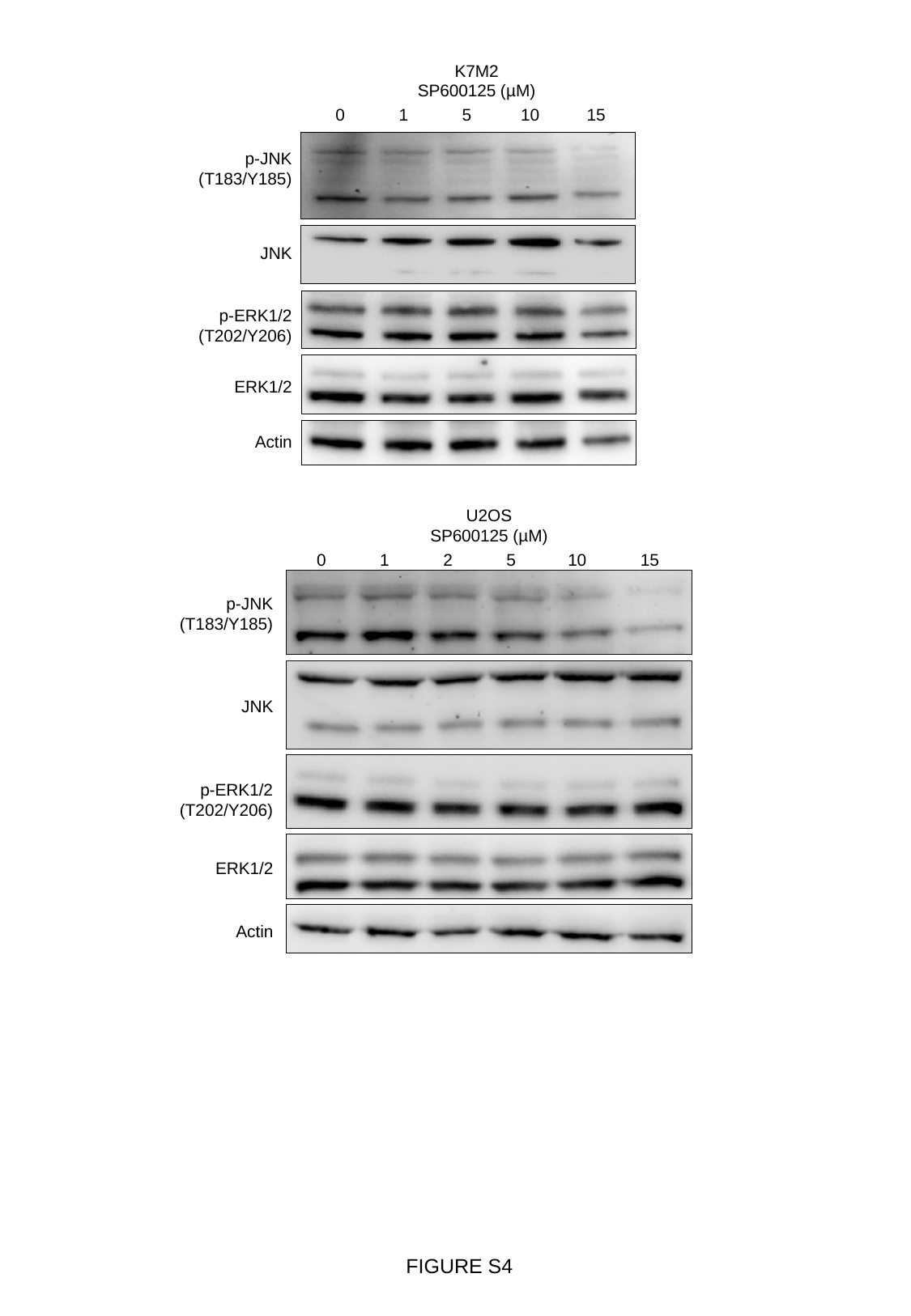

K7M2
SP600125 (µM)
0
1
5
10
15
p-JNK
(T183/Y185)
JNK
p-ERK1/2
(T202/Y206)
ERK1/2
Actin
U2OS
SP600125 (µM)
0
1
2
5
10
15
p-JNK
(T183/Y185)
JNK
p-ERK1/2
(T202/Y206)
ERK1/2
Actin
FIGURE S4

## Slide 2
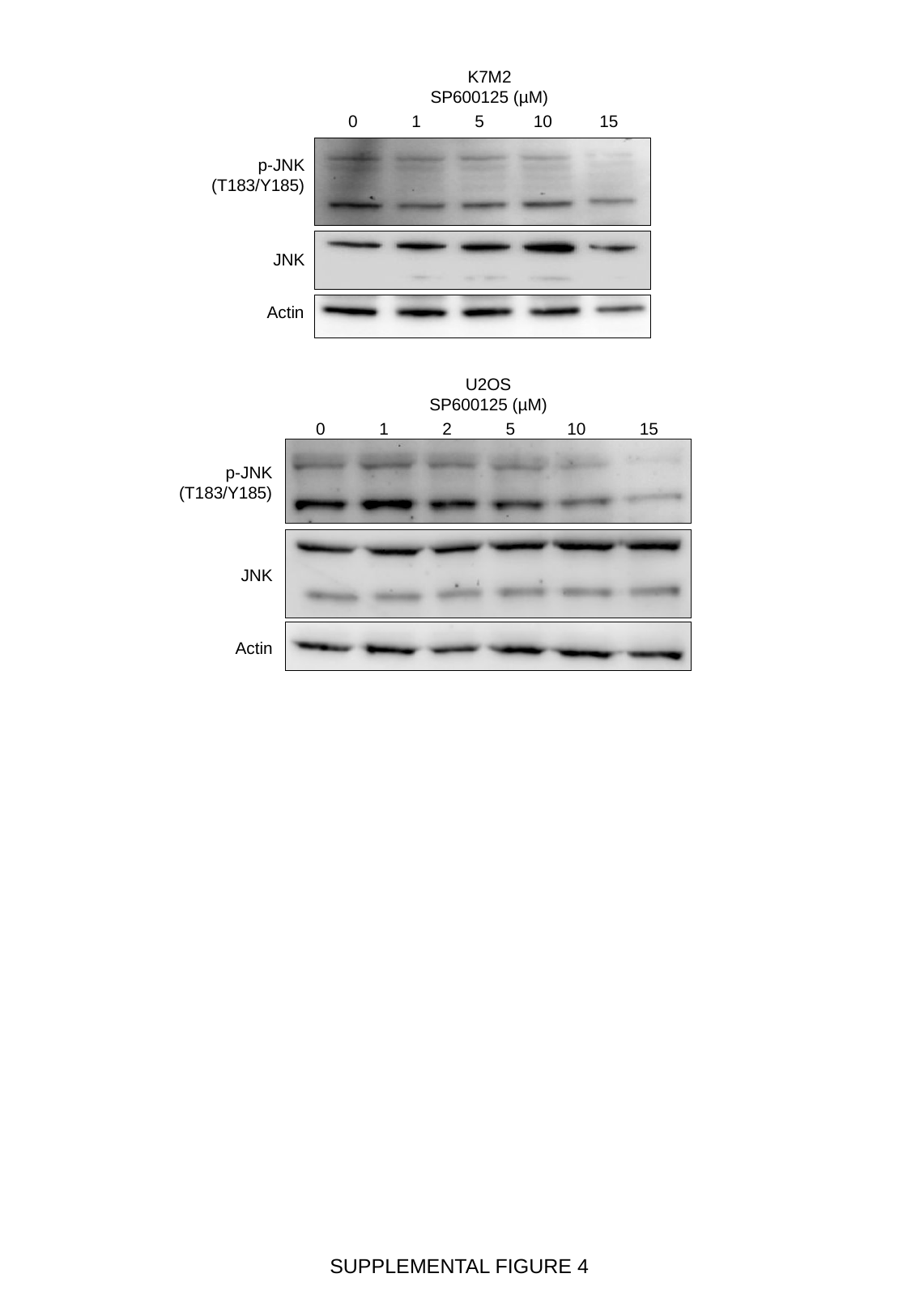

K7M2
SP600125 (µM)
0
1
5
10
15
p-JNK
(T183/Y185)
JNK
Actin
U2OS
SP600125 (µM)
0
1
2
5
10
15
p-JNK
(T183/Y185)
JNK
Actin
SUPPLEMENTAL FIGURE 4
