# Supplementary material for: CYR61 triggers osteosarcoma metastatic spreading via an IGF1Rβ-dependent EMT-like process
Source: BMC Cancer. 2019 Jan 14;19:62. doi: 10.1186/s12885-019-5282-4 (PMC6332662; doi:10.1186/s12885-019-5282-4)
Supplement: Supplementary file 5 — Table S1. Primer sequences for real time quantitative PCR (mouse). (DOCX 16 kb) [file 12885_2019_5282_MOESM5_ESM.docx]

Additional file 5: **Table S1.** Primer sequences for real time quantitative PCR (mouse)

| Target gene | Forward (5’-3’) | Reverse (5’-3’) |
| --- | --- | --- |
| 18S | CGGCTACCACATCCAAGGAA | GCTGGAATTACCGCGGCT |
| CYR61 | GGATCTGTGAAGTGCGTCCT | CTGCATTTCTTGCCCTTTTT |
| SNAIL | GTCTGCACGACCTGTGGAA | CAGGAGAATGGCTTCTCACC |
| SLUG | TGCAAGATCTGTGGCAAGG | CAGTGAGGGCAAGAGAAAGG |
| TWIST | AGCTACGCCTTCTCCGTCT | TCCTTCTCTGGAAACAATGACA |
| VIMENTIN | CCAACCTTTTCTTCCCTGAA | TGAGTGGGTGTCAACCAGAG |
| OCCLUDIN | GTCCGTGAGGCCTTTTGA | GGTGCATAATGATTGGGTTTG |
| DESMOPLAKIN | CCTGTGATGCGTATCAGAAAAG | TGTAAAGGGCTCGCATTTG |
| E-CADHERIN | GTTGCAGAAGGCGCTGTT | TGTTGACGTCATCGTCTGC |
| N-CADHERIN | GCCATCATCGCTATCCTTCT | CCGTTTCATCCATACCACAAA |
| MMP-2 | TAACCTGGATGCCGTCGT | TTCAGGTAATAAGCACCCTTGAA |
| MMP-3 | TTGTTCTTTGATGCAGTCAGC | GATTTGCGCCAAAAGTGC |
| MMP-9 | ACGACATAGACGGCATCCA | GCTGTGGTTCAGTTGTGGTG |
| MMP-14 | AACTTCGTGTTGCCTGATGA | CTTTGTGGGTGACCCTGACT |
| TIMP-2 | CGTTTTGCAATGCAGACGTA | GGAATCCACCTCCTTCTCG |
| IGF1 | AGCAGCCTTCCAACTCAATTAT | GAAGACGACATGATGTGTATCTTTATC |
| ZEB1 | AGGTGATCCAGCCAAACG | GGTGGCGTGGAGTCAGAG |
| MUCIN-1 | CTGTTCACCACCACCATGAC | CTTGGAAGGGCAAGAAAACC |
| ENTACTIN | AGCTTTGCTGGGTGGATG | ACTGGAGCCCTTCGAGAACT |
| ZO1 | ATGCAGACCCAGCAAAGG | TGGTTTTGTCTCATCATTTCTTCA |
