# Supplementary material for: CYR61 triggers osteosarcoma metastatic spreading via an IGF1Rβ-dependent EMT-like process
Source: BMC Cancer. 2019 Jan 14;19:62. doi: 10.1186/s12885-019-5282-4 (PMC6332662; doi:10.1186/s12885-019-5282-4)
Supplement: Supplementary file 6 — Table S2. Primer sequences for real time quantitative PCR (human). (DOCX 16 kb) [file 12885_2019_5282_MOESM6_ESM.docx]

Additional file 6: **Table S2.** Primer sequences for real time quantitative PCR (human)

| Target gene | Forward (5’-3’) | Reverse (5’-3’) |
| --- | --- | --- |
| 18S | CGGCTACCACATCCAAGGAA | GCTGGAATTACCGCGGCT |
| CYR61 | AAACCCGGATTTGTGAGGT | GCTGCATTTCTTGCCCTTT |
| SNAIL | GCTGCAGGACTCTAATCCAGA | ATCTCCGGAGGTGGGATG |
| SLUG | TGGTTGCTTCAAGGACACAT | GCAAATGCTCTGTTGCAGTG |
| TWIST | AGCTACGCCTTCTCGGTCT | CCTTCTCTGGAAACAATGACATC |
| VIMENTIN | TGGTCTAACGGTTTCCCCTA | GACCTCGGAGCGAGAGTG |
| OCCLUDIN | AGGAACCGAGAGCCAGGT | TGAGCAATGCCCTTTAGCTT |
| DESMOPLAKIN | CCAGAACTCGGACGGCTAC | ATCAAGCAGTCGGAGCAGTT |
| E-CADHERIN | TGGAGGAATTCTTGCTTTGC | CGCTCTCCTCCGAAGAAAC |
| N-CADHERIN | CTCCATGTGCCGGATAGC | CGATTTCACCAGAAGCCTCTAC |
| MMP-2 | ATAACCTGGATGCCGTCGT | AGGCACCCTTGAAGAAGTAGC |
| MMP-3 | CAAAACATATTTCTTTGTAGAGGACAA | TTCAGCTATTTGCTTGGGAAA |
| MMP-9 | GACAGGCAGCTGGCAGAG | CAGGGACAGTTGCTTCTGG |
| MMP-14 | CTGTCAGGAATGAGGATCTGAA | AGGGGTCACTGGAATGCTC |
| TIMP-2 | GAAGAGCCTGAACCACAGGT | CGGGGAGGAGATGTAGCAC |
| IGF1 | TGTGGAGACAGGGGCTTTTA | ATCCACGATGCCTGTCTGA |
| ZEB1 | AACTGCTGGGAGGATGACAC | TCCTGCTTCATCTGCCTGA |
| MUCIN1 | CCTGCCTGAATCTGTTCTGC | CATGACCAGAACCCGTAACA |
| ENTACTIN | CAGTTTTCAGATGAGGGAACG | AGGCCAGTTTCACAGTAGTTGAT |
| ZO1 | GGTCAGAGCCTTCTGATCATTC | CATCTCTACTCCGGAGACTGC |
